# Annotations

**Published:** 1907-06-29

**Authors:** 


### ANNOTATIONS.

### The Sleeping Sickness Conference.

The International Conference on Sleeping Sickness, which has been organised by His Majesty's Government, includes representatives of all the Powers who have interests in the tropical parts of the African Continent, where, as is now well-established, this terrible disease is responsible for an appalling mortality. The Conference is an attempt to secure co-operation and co-ordination among those who are working to obtain a scientific knowledge of the disease with a view to discover the therapeutic and prophylactic measures necessary to check its spread. In welcoming the foreign delegates to the Conference, the President, Lord Fitzmaurice, stated that in Uganda alone 200,000 out of a population of 300,000 in the infected area had fallen victims to this disease. He alluded to the tendency which the disease was showing to spread into new latitudes as a result of the more ready intercourse between different parts of Africa, and to the fact that these conditions were a direct consequence of the introduction of Euorpean administration. The discoveries that sleeping sickness is due to the *Trypanosoma Gambiense*, that the parasite is conveyed from one district to another by a species of tsetse fly, and that the distribution of this fly is limited to the neighbourhood of open water, are obviously facts of the first moment. These, as well as the demonstration of certain therapeutic measures and the methods of early diagnosis are the direct fruits of scientific study, and it may be confidently hoped that the outcome of the Conference will be a practical organisation by which the natives of tropical Africa may be relieved from a most terrible scourge.

### The Legal Position of a Medical Practitioner's Motor-Car.

A recent judgment by the King's Bench Division of the High Court of Justice is not without interest to members of the medical profession. The matter in dispute was whether rent could be charged for water, supplied by a public authority, and used by a medical practitioner for washing a motor-car employed, at least in part, for professional purposes. A complaint to recover such a charge was brought by the Corporation of Harrogate against Dr. Ian Donald Mackay, and was first heard by the local magistrates, who found in favour of Dr. Mackay, but stated a case for the Court of Appeal. It appears that under a local Act of Parliament the Corporation has power to supply water for "other than domestic purposes" on such terms as they may think fit, and that in the Waterworks Act of 1863 it is enacted that "a supply of water for domestic purposes shall not include a supply of water for cattle or for horses, or for washing carriages where such horses or carriages are kept for sale or hire or by a common carrier." Thus, the question at issue was whether water used in the washing of a medical man's motor-car was or was not used "for a domestic purpose." For the Corporation it was argued that as the motor-car was "used for a business purpose" water supplied for use on such a car was supplied for "other than domestic purposes," and could, therefore, be charged at a special rate. On the other hand, it was claimed by the practitioner that the water used in washing the car was employed in a "domestic purpose," as he did not keep his car "for sale or hire," and he was not "a common carrier." The Lord Chief Justice, in giving judgment in favour of Dr. Mackay, said that it had previously been decided that the washing of a carriage was a domestic purpose, and that this was only limited where a carriage was "kept for sale or hire." To extend such a limitation to a carriage used by a professional man was not an arguable position. Mr. Justice Darling and Mr. Justice A. T. Lawrence concurred, and the appeal, therefore, was dismissed. We congratulate Dr. Mackay on his courage and success.

### Examinations and Research.

The Federal Conference on Education, which recently met in London, can hardly fail to arouse and stimulate at least some measure of public interest in questions that very closely concern the national well-being. The work of our Universities ought to be a matter worthy of the attention of all who reflect seriously on the problems of the immediate future. It is gratifying to find that the provision of opportunities for post-graduate study and research received general recognition as one of the most important of the claims which fall within the area of academic responsibility. The medical profession has a most direct interest in this aspect of University duty, and it is more than ready to provide sympathy and practical help in support of such a demand. In not a few medical centres in recent years active expression has been given to this view in spite of the absence of official initiative. But it may be doubted whether some of the most ardent advocates of this function of University organisations do not sometimes forget that cultivation in the direction of their ambitions does not exhaust the scope of University duties. Not every student, nor indeed the majority of students, can with any advantage be directed into the field of original research. Special abilities, or at any rate special aptitudes, are required, and these are, and probably always will be, the possession of a select few. There remain the great majority, possessing average capacity and easily capable of being trained into competent workers and reasonable thinkers, and for these our Universities ought to provide efficient training. By all means let original research be cultivated and developed. But this should not mean that other claims may be disregarded. There is just now something like a dead set against examination and preparation for examination. The virtues of the system may have been exaggerated, but rightly used, examinations may be made effective educational instruments, and the teaching which is a necessary preliminary to them is as much the duty of an academic organisation as is the development of research.

